# Imiquimod‐induced apoptosis of melanoma cells is mediated by ER stress‐dependent Noxa induction and enhanced by NF‐κB inhibition

**DOI:** 10.1111/jcmm.12718

**Published:** 2015-11-18

**Authors:** Abdelouahid El‐Khattouti, Denis Selimovic, Matthias Hannig, Erin B. Taylor, Zakaria Y. Abd Elmageed, Sofie Y. Hassan, Youssef Haikel, Emad Kandil, Martin Leverkus, Robert T. Brodell, Mosaad Megahed, Mohamed Hassan

**Affiliations:** ^1^ Cancer InstituteUniversity of Mississippi Medical CenterJacksonMSUSA; ^2^ Clinic of Operative Dentistry, Periodontology and Preventive DentistryUniversity Hospital of SaarlandHomburg/SaarGermany; ^3^ Department of Physiology and BiophysicsUniversity of Mississippi Medical CenterJacksonMSUSA; ^4^ Department of SurgeryTulane University School of MedicineNew OrleansLAUSA; ^5^ Clinic of DermatologyUniversity Hospital of AachenAachenGermany; ^6^ Institut National de la Santé et de la Recherche MédicaleUniversity of StrasbourgStrasbourgFrance; ^7^ Department of Operative Dentistry and EndodonticsDental FacultyUniversity of StrasbourgStrasbourgFrance; ^8^ Department of DermatologyUniversity of Mississippi Medical CenterJacksonMSUSA; ^9^ Department of PathologyUniversity of Mississippi Medical CenterJacksonMSUSA

**Keywords:** melanoma, imiquimod, apoptosis, ER stress, NF‐κB

## Abstract

Melanoma is characterized by dysregulated intracellular signalling pathways including an impairment of the cell death machinery, ultimately resulting in melanoma resistance, survival and progression. This explains the tumour's extraordinary resistance to the standard treatment. Imiquimod is a topical immune response modifier (imidazoquinoline) with both antiviral and antitumour activities. The mechanism by which imiquimod triggers the apoptosis of melanoma cells has now been carefully elucidated. Imiquimod‐induced apoptosis is associated with the activation of apoptosis signalling regulating kinase1/c‐Jun‐N‐terminal kinase/p38 pathways and the induction of endoplasmic stress characterized by the activation of the protein kinase RNA‐like endoplasmic reticulum kinase signalling pathway, increase in intracellular Ca^2+^ release, degradation of calpain and subsequent cleavage of caspase‐4. Moreover, imiquimod triggers the activation of NF‐κB and the expression of the inhibitor of apoptosis proteins (IAPs) such as, X‐linked IAP (XIAP) together with the accumulation of reactive oxygen species (ROS). Also, imiquimod triggers mitochondrial dysregulation characterized by the loss of mitochondrial membrane potential (Δψm), the increase in cytochrome c release, and cleavage of caspase‐9, caspase‐3 and poly(ADP‐ribose) polymerase (PARP). Inhibitors of specific pathways, permit the elucidation of possible mechanisms of imiquimod‐induced apoptosis. They demonstrate that inhibition of NF‐kB by the inhibitor of nuclear factor kappa‐B kinase (IKK) inhibitor Bay 11‐782 or knockdown of XIAP induces melanoma apoptosis in cells exposed to imiquimod. These findings support the use of either IKK inhibitors or IAP antagonists as adjuvant therapies to improve the effectiveness topical imiquimod in the treatment of melanoma.

## Introduction

Melanoma is an aggressive form of skin cancer with high metastatic potential and a notoriously high resistance to cytotoxic agents. Although many chemo‐ and immune‐based therapies for melanoma treatment have been evaluated in clinical trials, most of them failed to show significant benefit in melanoma patients 1, 2, 3.

Several mechanisms, including dysregulation of apoptosis, drug transport, detoxification, methylation and enhanced DNA repair have been described 4. Similar to other malignant tumours, melanoma resistance to chemotherapy has been attributed to the elevated expression of Mcl‐1, XIAPs, or alterations of NF‐κB whose down‐regulation or inhibition sensitize melanoma cells to anticancer agent‐induced apoptosis 5, 6, 7. Recognizing these difficulties, novel therapies are needed to bypass the intrinsic and acquired pathways that permit malignant melanoma to resist drug induced cell death. Therefore, the mode of action of already clinically proven therapeutics in clinical trials and/or clinical use may help to further improve the treatment outcome of melanoma and/or minimize its resistance development 8.

Imiquimod belongs to the class of Toll‐like receptor (TLR) agonists with high affinity to TLR7 9. In clinical trials, imiquimod has demonstrated significant efficacy in the treatment of non‐melanoma (keratinocyte‐based skin cancers) as well as malignant melanoma 10, 11, 12. Also, the ability of imiquimod to exert its biological activity *via* modulation of variable signalling pathways has been demonstrated in several studies 13, 14.

Toll‐like receptors belong to a family of endosomal nucleic acid‐sensing molecules with pleiotropic cellular functions 15, 16. As TLR7 is mainly localized on the endoplasmic reticulum (ER), its function seems to be mediated by ER stress and/or pro‐inflammatory‐associated pathways 17, 18.

Endoplasmic reticulum plays an important role in the maintenance of intracellular calcium homoeostasis, protein synthesis, post‐translational modifications and proper folding of proteins as well as their sorting and trafficking. Alterations in calcium homoeostasis and accumulation of unfolded proteins cause ER stress 19. A variety of agents including chemical toxicants, oxidative stress and inhibitors of protein glycosylation have been investigated for their ability to trigger ER stress. This involves the induction and translocation of BH3‐only pro‐apoptotic Bcl‐2 proteins such as Noxa to ER membranes 20. Nevertheless, the relative contribution of ER stress signalling in the modulation of imiquimod‐induced apoptosis is less obvious to date. Based on the nature of TLR7 as an endoplasmic receptor, its contribution to imiquimod‐induced ER stress and its subsequent consequences are expected. Of note, ER stress‐ associated pathways are linked to mitochondrial dysregulation‐dependent mechanisms in most tumour cells 21, 22, 23.

The nuclear transcription factor, NF‐κB, plays an important role in carcinogenesis as well as in the regulation of immune and inflammatory responses 24, 25, and NF‐κB activity has been implicated in tumour progression and therapeutic resistance of melanoma 26. Activation of NF‐κB mediates the expression of diverse target genes that promote cell proliferation, regulate apoptosis, facilitate angiogenesis and stimulate invasion and metastasis 27, and thus serves as a key transcriptional regulator of anti‐apoptotic and antioxidant molecules. In most cases, the death protective NF‐κB activity depends on its ability to transactivate gene expression; however, some exceptions to this rule have been reported 28, 29, 30. The suppression of apoptosis by NF‐κB relies on activating a set of transcriptional target genes, whose products can block different steps of extrinsic and/or intrinsic death‐signalling cascades. These targets include the IAPs XIAP, c‐IAP1 and c‐IAP2, which have been implicated in the prevention of pro‐caspase‐9 activation and blocking caspase‐3 and‐7 activity 31, 32. XIAP has also been proposed to block cell death as a result of persistent activation of c‐Jun‐N‐terminal kinase (JNK) 33. Other studies indicated that XIAP inhibits JNK activation and apoptosis induced by transforming growth factor (TGF)‐β1, *via* the induction of the ubiquitination and degradation of the TGF‐beta1‐activated kinase 34. Another member of the IAP family, Survivin, is also regulated by NF‐κB 35, 36.

In this study, the molecular mechanisms of imiquimod‐induced cell death of melanoma cells are elucidated. These findings support the development of adjuvant therapeutic strategies using IKK inhibitors or IAP antagonists to enhance the effectiveness of imiquimod in the treatment of malignant melanoma.

## Materials and methods

### Cell culture, inhibitors and treatment

The human melanoma cell lines BLM (highly invesive melanoma cell line) and MV3 were obtained from Dr. van Muijen, Pathology Department, University Hospital Nijmegen St. Radboud, and Nijmegen, Netherlands. Up on receiving the cell lines, we tested them for mycoplasma using a mycoplasma detection kit (Qiagen, Hilden, Germany). In addition, the cell lines were re‐authenticated every 6 months using PowerPlex^®^ 18D System; Promega, Madison, WI, USA). All cell lines were grown in DMEM supplemented with 10% heat‐inactivated foetal bovine serum, 2 mM glutamine and 1% antibiotic solution at 37°C in a humidified atmosphere of 5% CO_2_. The treatment of the cells with imiquimod (gift of 3M, St. Paul, MN, USA) was dissolved in 25% HCl and the treatment of the cells was made at a concentration of 50 μg/ml for time periods up to 48 hrs, whereas the control cells were treated with 25% HCl. 4‐phenylbutyrate (4‐PBA), the inhibitor of ER stress, was obtained from Sigma‐Aldrich Chemie Gmbh, Munich, Germany and was used at a concentration of 200 μM. 5,5′,6,6′‐tetrachloro‐1,1′,3,3′‐tetraethylbenzimidazolylcarbocyanine iodide (JC‐1) was purchased from Biotrend ChemiKalien (Cologne, Germany).

### MTT colorimetric assay

Determination of cell viability was performed with the MTT assay as described previously 37.

### Detection of apoptosis using the annexin V/propidium iodide

The analysis of imiquimod‐induced apoptosis of BLM and MV3 cells by flow cytometry using annexin V‐FITC/propidium iodide (PI; Vybrant; Invitrogen, Karlsruhe, Germany) for 15 min. at room temperature and protected from light is carried out as described previously 20.

### Measurement of mitochondrial membrane potential (ΔΨm) using JC‐1

BLM and MV3 cells were stained with 10 μM JC‐1 for 30 min. at room temperature in the dark. The intensities of green (520–530 nm) and red fluorescence (>550 nm) of 50,000 individual cells were analysed by flow cytometry as described previously 20.

### Immunoblot analysis

Immunoblot analysis was performed according to the standard procedures using the following antibodies and dilutions: Anti‐apoptosis signalling regulating kinase1 (ASK1; Sc‐7931), 1:500; anti‐ p‐ASK1 (Thr 845, Sc‐109911), 1:1000; anti‐ JNK (Sc‐474), 1:1000; anti‐ p‐JNK (SC‐6254), 1:1000; anti‐ p38 (Sc‐535), 1:1000; anti‐ p‐p38 (SC‐7973), 1:1000; anti‐ Noxa (SC‐2697), 1:1000; anti‐Mcl‐1 (SC‐20679), 1:500; anti‐ Actin (Sc‐1615), 1:5000; anti‐ Tom20 (Sc‐11415), 1:100; anti‐ Bap31 (Sc‐17764), 1:500; anti‐ IRE1α (Sc‐20790), 1:500; anti‐ protein kinase RNA‐like endoplasmic reticulum kinase (PERK; SC‐9477), 1:1000; anti‐ p‐ERK (SC‐32577), 1:1000; anti‐ calpain 1 (Sc‐7530), 1:1000; anti‐ caspase‐4 (Sc‐1229), 1:1000; anti‐ ATF4 (SC‐200), 1:1000; anti‐ XIAP (SC‐11426), 1:1000; anti‐ IκB‐α (sc‐203), 1:500; anti‐ p‐IκB‐α (sc‐8404), 1:500 (from Santa Cruz Biotechnology Inc., Santa Cruz, CA, USA); anti‐ p‐IRE1α (PA1‐16927; Thermo Scientific, FL 33407, USA), 1:1000; anti‐ CHOP (#28950, 1:1000; anti‐ caspase 3 (#7190), 1:1000; anti‐ caspase 9 (#9501), 1:1000; anti‐ PARP (#9542), 1:500; anti‐ Erk1/2 (#9102), 1:1000; anti‐ p‐Erk1/2 (#9101), 1:1000 (from Cell Signalling Technology Inc., Danvers, MA, USA); anti‐ p‐IRE1 (ab48187), 1:1000; anti‐ p‐ATF‐4 (ab28830), 1:1000; anti‐GRP78 (ab108613), 1:500 (from Abcam, Massachusetts, USA).

### Preparation of nuclear extracts

Nuclear extracts were prepared from treated and untreated BLM and MV3 cells as described 37. Briefly, the cells were washed with ice‐cold PBS buffer and harvested by adding 500 μl of buffer a [20 mM Hepes, pH 7.9; 10 mM NaCl, 0.2 mM ethylenediaminetetraacetic acid (EDTA) and 2 mM 1,4‐dithiothreitol (DTT)] containing protease inhibitors and incubated on ice for 10 min. The supernatant was discarded after centrifugation at 14,000 r.p.m. for 3 min. The pellet was resuspended in 50 μl of buffer C (20 mM Hepes, pH 7.9; 420 mM NaCl, 0.2 mM EDTA; 2 mM DTT; 1 mM Na_3_VO_4_, 25% glycerol) containing protease inhibitors at 4°C for 20 min. prior to centrifugation at 14,000 r.p.m. for 3 min. The supernatant was collected without disturbing the pellet and stored at −80°C until use.

### Electrophoretic mobility shift assay

The details of electrophoretic mobility shift assay (EMSA) have been described elsewhere 37. The double‐stranded synthetic oligonucleotides carrying a defined binding site for AP‐1, ATF‐2, p53, NF‐κB and ELK‐1 (Santa Cruz Biotechnology Inc.), ATF‐3 (5′‐GTGACGT[AC][AG]‐3′) were end‐labelled with [γ‐^32^P] ATP (Hartmann Analytika, Munich, Germany) in the presence of T4 polynucleotide kinase (Genecraft, Munster, Germany). Around 4 μg of nuclear extracts were bound to a labelled probe in a total volume of 30 μl for 30 min. at room temperature in binding buffer (10 mM Tris, pH 7.5; 50 mM NaCl, 1 mM EDTA; 1 mM MgCl_2_; 0.5 mM DTT and 4% glycerol). The specificity of the binding was analysed by competition with an unlabelled oligonucleotide assay. The competition assay was performed in the same manner except that unlabelled probes containing oligonucleatide sequences (binding sites) were incubated with nuclear extracts for 20 min. at room temperature before adding the labelled probes. Electrophoresis was performed for 3 hrs at 100 V in 0.5 X Tris‐borate‐EDTA running buffer at room temperature. The dried gel was visualized by exposure to high performance autoradiography film.

### Staining of intracellular calcium

The cytosolic Ca^2+^ signals of imiquimod‐treated BLM and MV3 cells were monitored by flow cytometry using Ca^2+^ ‐sensitive dye‐Fluo3‐AM (Invitrogen) at a concentration of 4 μM for 30 min. at room temperature as described previously 19.

### Detection of ROS

The detection of ROS was performed by staining cells with DHR 123 (Sigma‐Aldrich) and analysed by flow cytometry as described previously 19.

### Immunofluorescence staining

BLM cells were allowed to grow for 24 hrs before the exposure to imiquimod for 24 hrs. Then the cells were subjected to immunofluorescence staining as described 20. Primary antibodies, anti‐Noxa (SC‐2697), 1:200; anti‐Tom20 (Sc‐11415), 1:200; anti‐Bap31 (Sc‐17764), 1:200 (each Santa Cruz Biotechnology Inc.) were allowed to bind for 2 hrs at room temperature. Subsequently, the cells were washed three times in PBS and incubated with Alexa Flour‐labelled secondary antibodies for 2 hrs at room temperature. After an additional three washes in PBS, the cells were mounted using DAKO mounting medium. Photomicrographs were taken on a Leica fluorescence microscope (Leica, Wetzlar, Germany).

### Preparation of mitochondrial and ER fractions

The preparation of mitochondrial and ER fractions were performed as described 19. Briefly, imiquimod‐ treated and untreated cells (BLM and MV3) were scraped off with 5 ml of phosphate‐buffered saline. Harvested cells were precipitated by centrifugation at 600 × g for 5 min., washed, resuspended and homogenized. The homogenized cells were then centrifuged at 600 × g for 5 min., and subsequently, the supernatant was layered over a discontinuous gradient of 40% and 60% sucrose in HE buffer (3 and 1 ml respectively). After centrifugation at 100,000 × g for 3 hrs, both ER and mitochondrial fractions were collected and subsequently pelleted by centrifugation at 100,000 × g for 1 hr. The pellet of mitochondria was resuspended in buffer D (25 mM potassium phosphate and 5 mM MgCl_2_, pH 7.2). Aliquots of both ER and mitochondria were precipitated using 10% trichloroacetic acid (TCA) and subjected to SDS‐PAGE and immunoblotting using specific antibodies for mitochondria and ER.

### RNA interference

The knockdown of XIAP in melanoma cells was performed with XIAP‐specific siRNA (I #6446; Cell Signalling Technology), whereas the knockdown of Noxa was performed by NOXA siRNA (sc‐37305; Santa Cruz Biotechnology, Inc.), using lipfectamine 2000 as described 37.

### Plasmids, transfections and luciferase reporter assay

NF‐κB luciferase reporter assay was performed with the pNF‐κB‐Luc plasmid (Strategene, LaJolla, CA, USA). The transfection of BLM and MV3 cells with the pNF‐κB‐Luc plasmid was performed as described 38. Whereas, the NF‐κB‐dependent luciferase activity was measured using the Dual Luciferase Reporter Assay system (Promega). Briefly, Melanoma cells (1 × 10^5^ cells/well) were seeded in a 96‐well plate for 24 hrs. The cells were then transfected with plasmids for each well and then incubated over a transfection period of 24 hrs. After that, the cell culture medium was removed and replaced with fresh medium containing imiquimod (50 μg/ml). Forty‐eight hours later, the Luciferase activity was determined in Microlumat plus luminometer (EG & G Berthold, Bad Wildbad, Germany) by injecting 100 μl of assay buffer containing luciferin and measuring light emission for 10 sec. Cotransfection with pRL‐CMV (Promega), which expresses Renilla luciferase, was performed to enable normalization of data for transfection efficiency.

### RT‐PCR analysis

RT‐PCR was performed to determine NF‐κB target gene expression following the treatment of melanoma cells with imiquimod. In brief, Melanoma cells were treated with imiquimod for 48 hrs. Cells were harvested and washed twice with ice‐cold PBS, and then total RNA was isolated from cells using RNeasy Mini kits according to the manufacturer's instructions (Qiagen). Complementary DNA was synthesized from1 μg of total RNA in a 20 μl reverse transcription reaction mixture according to the manufacturer's protocol (Gencraft, Munster, Germany). The following primer pairs were used for RT‐PCR amplification: human interleukin‐8 (IL‐8), 5′‐GAT GCC AGT GAA ACT TCA AGC‐3′ and 5′‐CTG GCA TTT GTG GTT GGG TCA‐3′; human IL‐6 5′‐TCT CAG CCC TG A GAA AGG AGA‐3′ Glyceraldehyde‐3‐phosphate dehydrogenase (GAPDH) was carried out (forward primer: 5′‐ACC AGC GAC ACC CAC TCC TC‐3′ reverse primer: 5′‐GGA GGG GAG ATT CAG TGT GGT‐3′ was used as the housekeeping gene control. The reaction was performed in 25 μl (total volume) mixtures containing primers at a concentration of 100 nM. The reaction conditions consisted of 5 min. at 94°C, and then 35 cycles of 30 sec. at 94°C, 30 sec. at 58°C, and 30 sec. at 72°C. The final extension was done at 72°C for 10 min. PCR products were run on 3% agarose gel and then stained with ethidium bromide, stained bands were visualized under UV light and photographed.

### Statistical analysis

Data were expressed as mean ± SD. Statistical comparisons were performed with one‐way anova followed by the Scheffe's test. Statistical differences between two groups were determined using the unpaired Student's *t*‐test. A *P* < 0.05 was considered as statistically significant. All statistical analysis was performed with SPSS statistical software (version 16.0; SPSS, Chicago, IL, USA).

## Results

### Sensitivity of human melanoma cells to imiquimod correlates with the induction of ER stress‐dependent pathways

**Figure 1:**
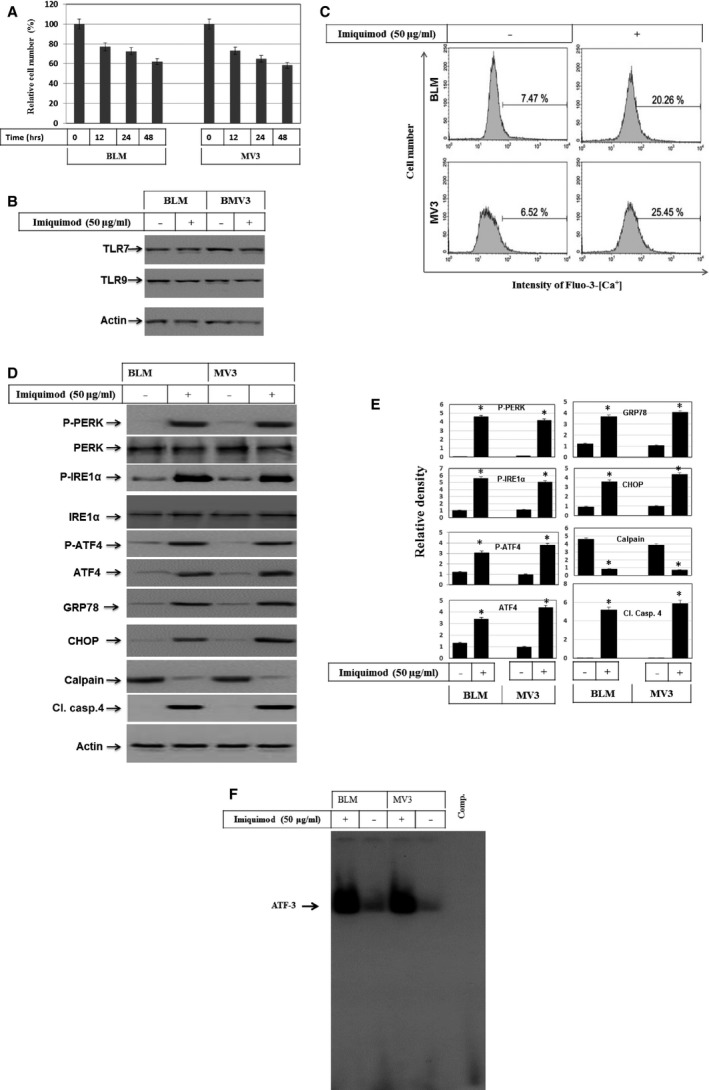
(**A**) MTT assay demonstrates imiquimod‐induced time dependent growth inhibition of melanoma cell lines BLM and MV3. The cells were treated with imiquimod (50 μg/ml) for the indicated time periods up to 48 hrs. The results are the mean of three independent experiments performed in quadruplicate. (**B**) Western blot demonstrated the expression of TLR‐7 and TLR9 before and after the exposure of melanoma cells to imiquimod for 48 hrs. Actin was used as an internal control for loading and transfer. (**C**) Flow cytometry analysis using Ca^2+^‐sensitive dye‐Fluo3‐AM staining demonstrates intracellular Ca^2+^ release following the exposure of melanoma cell BLM to imiquimod for 48 hrs. (**D**) Western blot analysis demonstrates the phosphorylation and the expression levels of PERK, IRE1α, and ATF‐4, the expression of GRP78 and CHOP, and the degradation of calpain and cleavage of caspase‐4 in control and imiquimod‐treated melanoma cells. Actin was used as an internal control for loading and transfer. (**E**) Densitometric analyses are presented as the relative ratio of phospho‐PERK, phospho‐IRE1α, phospho‐ATF4, ATF4, GRP78, CHOP, Calpain and Cl. casp.‐4 to actin. Bars represent mean ± SD from three blots. *P < 0.05 *versus* control. (**F**) EMSA demonstrates the enhancement of the DNA‐binding activity of the transcription factor ATF‐3 in response to the treatment of melanoma cells with imiquimod for 48 hrs. Data are representative of three independent experiments.

The effect of imiquimod on melanoma cells was assessed using the recommended concentration as determined by Schon *et al*. 39 Accordingly, the melanoma cell lines BLM and MV3 were treated with imiquimod (50 μg/ml) for time intervals up to 48 hrs and cell viability was performed by MTT assays. Imiquimod‐induced cell death was observed as early as 12 hrs (~20%) after stimulation with further increase up to 48 hrs (~40%) in both cell lines examined (Fig. 1A). We also examined whether imiquimod influences the expression of TLR7 and TLR9. Western blot analysis (Fig. 1B), however, revealed no marked alteration of protein expression of either TLR7 or TLR9.

Based on the fact that TLR7 and 9, the target receptors of imiquimod, reside within the ER, we next asked, whether the exposure of melanoma cells to imiquimod triggers ER stress, and if so, what is the result of ER stress on calcium homoeostasis? Next, we measured the intracellular calcium release following exposure of cells to imiquimod for 48 hrs by flow cytometry following staining with the Ca^2+^ ‐sensitive dye‐Fluo3‐AM. Flow cytometry analysis demonstrated the increase in intracellular calcium release (~3‐ to 4‐fold) in both melanoma cell lines BLM and MV3 (Fig. 1C). We then investigated whether imiquimod‐induced ER stress of the two melanoma cell lines is associated with activation of PERK, inositol‐requiring‐1α (IRE1α), activating transcription factor 4 (ATF‐4), the unfolded protein response (UPR) regulator (GRP78), C/EBP‐homologous protein (CHOP), calpain and caspase‐4 or ATF‐3. Around 48 hrs after stimulation with imiquimod, cells were harvested and total cellular lysates and nuclear extracts were utilized for Western blot analysis and EMSA respectively. Exposure of melanoma cells to imiquimod induces the phosphorylation of PERK and IRE1α, and both the expression and phosphorylation of ATF‐4 together with expression of GRP78 protein (Fig. 1D and E). Of note, PERKα or IRE1α basal expression was unchanged (Fig. 1D and E). Also, imiquimod treatment was found to induce the expression of the CCAAT‐enhancer‐binding protein homologous protein (CHOP) together with degradation of calpain and cleavage of caspase‐4 (Fig. 1D and E). This prompted us to investigate the DNA‐binding activity of the transcription factor ATF‐3, the substrate of ATF‐4 by EMSA. Electrophoretic mobility shift assay demonstrated increased DNA‐binding activity of ATF‐3 in response of melanoma cells to imiquimod treatment (Fig. 1F).

### Imiquimod‐induced apoptosis of melanoma cells is associated with mitochondrial dysregulation

**Figure 2:**
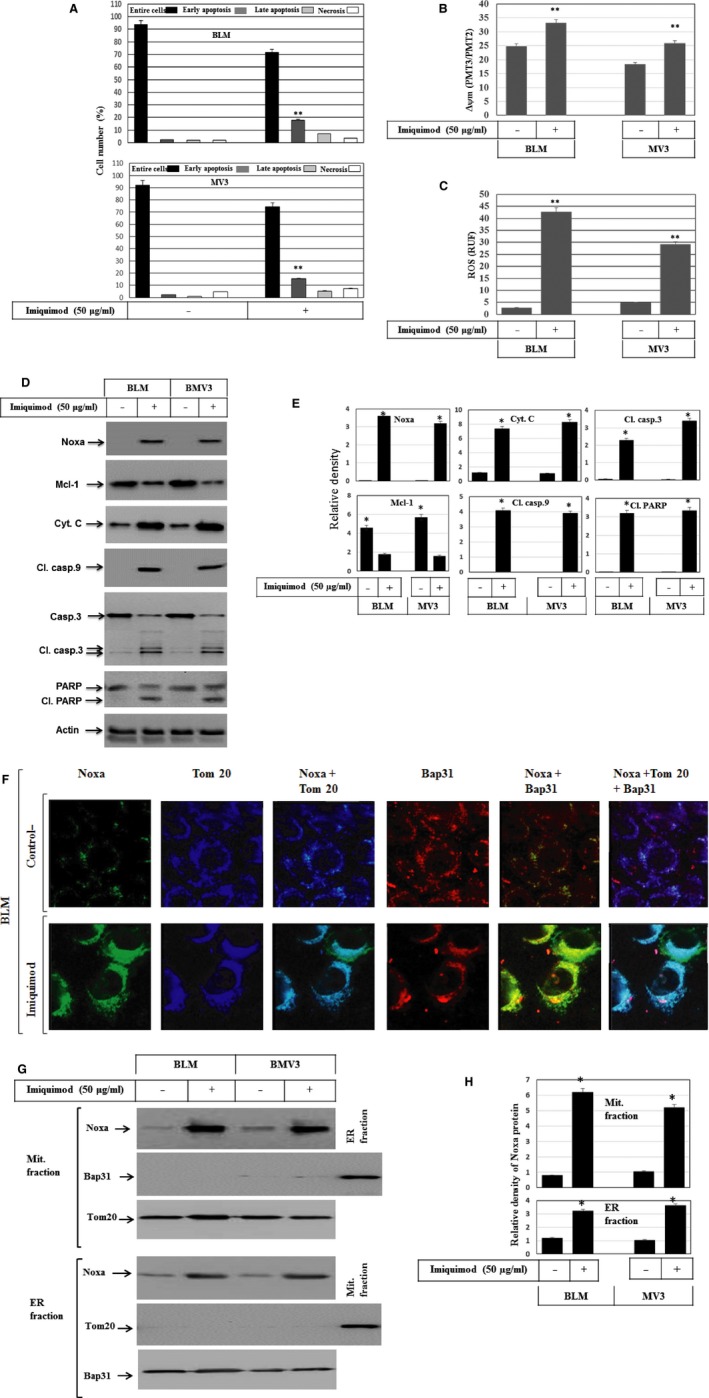
**(A)** Detection of apoptosis in melanoma cells by flow cytometry analysis using annexin/PI staining following the incubation of BLM and MV3 cell lines with imiquimod for 48 hrs. Data are representative of three independent experiments. **(B)** Loss of mitochondrial membrane potential in BLM and MV3 melanoma cells. BLM and MV3 melanoma cells were treated with imiquimod and stained with JC‐1 followed by flow cytometry analysis. Melanoma cells with intact mitochondria displayed high red and high green fluorescence and appeared in the upper right quadrant of the scatter plots. In contrast, cells that had lost their mitochondrial membrane potential displayed high green and low red fluorescence and appeared in the lower right quadrant. Data were represented as mean ± SD of three independent experiments. **P < 0.01, significantly different when compared with control cells. **(C)** Flow cytometry analysis demonstrates imiquimod‐induced ROS accumulation in BLM and MV3 using dihydrorhodamine (DHR 123). Data represented as mean ± SD of three independent experiments. **P < 0.01, significantly different when compared with control cells. **(D)** Western blot analysis demonstrates the induction of Noxa expression and the suppression of Mcl‐1, the increase of cytochrome c release (as assessed in the cytosol), cleavage of caspase‐9, caspase‐3 and PARP in response to the treatment of BLM and MV3 cells with imiquimod for 48 hrs. Actin was used as an internal control for loading and transfer. Data are representative of three independent experiments. **(E)** Analyses of band intensity on films are presented as the relative ratio of Noxa, Cyt. C, Cl. casp.3, Mcl‐1, Cl. casp.9 and Cl. PARP to actin. Bars represent mean ± SD from three blots. *P < 0.05 *versus* control. **(F)** Subcellular localization of Noxa protein to mitochondria and endoplasmic reticulum. Immune fluorescence: BLM cells were treated with imiquimod for 48 hrs before the staining with anti‐ Noxa, Tom20 (mitochondrial marker), Bap31 (ER marker). The subcellular localization of Noxa (green) to mitochondria (blue) and the overlay of Noxa with Tom20 staining demonstrates the localization of Noxa to mitochondria (turquoise), when compared to the control cells, and the subcellular localization of Noxa (green) to ER (red) and the overlay of Noxa with Bap31 staining demonstrates the localization of Noxa to ER (yellow), when compared to control cells, in addition to the overly of Noxa, Tom20 and Bap31 show the subcellular localization of Noxa to both mitochondria and ER. **(G)** Western blot analysis of mitochondrial (Mit. fraction) and ER fraction (ER fraction) isolated from both BLM and MV3 cells following the treatment with imiquimod for 48 hrs. The detection of Noxa in mitochondria and ER fractions derived from BLM and MV3 after the exposure to imiquimod confirms the localization of Noxa protein to both mitochondria and ER. The purity of the fractions was verified by the detection of the mitochondrial protein Tom20 and the ER marker Bap31in the corresponding fractions. Data are representative of three independent experiments. **(H)** Analyses of band intensity on films are presented as the relative ratio of Noxa to Tom20 in mitochondrial fraction and Noxa to Bap31 in ER fraction. Bars represent mean ± SD from three blots. *P < 0.05 *versus* control.

Next, we assessed the cytotoxicity of imiquimod for melanoma cells. Imiquimod‐induced apoptosis was analysed by flow cytometry using FITC annexin‐V and PI. As visualized in Figure 2A, induction of apoptosis of both BLM and MV3 cells in response to the treatment with imiquimod was detected. The loss of mitochondrial membrane potential (Δψm) in both melanoma cell lines BLM and MV3 was determined by JC‐1 staining using flow cytometry analysis before and after exposure of cells to imiquimod. Imiquimod induced a rapid loss of Δψm (Fig. 2B), suggesting an important role for mitochondria in the elicitation of imiquimod‐induced apoptosis. Considering the fact that excessive ROS cause apoptosis through numerous mechanisms including the loss of Δψm 40, we examined whether imiquimod‐induced mitochondrial damage is associated with the accumulation of ROS. Both melanoma cell lines were treated with imiquimod and ROS accumulation was assessed by flow cytometry analysis using dihydrorhodamine (DHR 123). Figure 2C demonstrates that imiquimod treatment of melanoma cells increases the accumulation of ROS, suggesting a contribution role for ROS in the regulation of imiquimod‐induced apoptosis of melanoma cells. Complementing these findings, we aimed to investigate the expression of pro‐ and anti‐apoptotic proteins such as Noxa and Mcl‐1, respectively, as well as caspase activation. Figure 2D and E demonstrate the induction of Noxa, degradation of Mcl‐1, increased cytochrome c release and cleavage of caspase‐9, caspase‐3 and PARP, during the treatment with imiquimod, suggesting the contribution of mitochondrial dysregulation‐dependent pathways in the modulation of imiquimod‐induced apoptosis of melanoma cells. To elucidate the role of Noxa more firmly, we next addressed the role of Noxa in the execution of imiquimod‐induced mitochondrial dysregulation. To do so, we investigated the subcellular localization of Noxa to either mitochondria or ER in BLM cells. Cells were treated with imiquimod for 48 hrs, fixed and subsequently subjected to staining with antibodies against Noxa, and Tom20 (specific marker for mitochondria), and Bap31 (specific marker for ER). Fluorescence microscopy of three independent experiments suggested a localization of Noxa protein to both mitochondria and ER (Fig. 2F). When we prepared subcellular fractions of mitochondria and ER in both BLM and MV3 cells, we could detect Noxa in both mitochondrial and ER fractions derived from imiquimod‐treated cells, but not in the control cells (Fig. 2G and H). To rule out contamination of the fractions, we assessed the purity of both mitochondrial and ER fractions by either Tom20 (marker for mitochondria) or Bap31 (marker of ER). In conclusion, these data suggest that imiquimod induced mitochondrial dysregulation and concomitant ER stress. We suggest that the induction and translocation of Noxa to mitochondria and ER represents a critical step during imiquimod‐induced apoptosis.

### Activation of MAP kinase signalling pathways in response to the treatment of melanoma cells with imiquimod

**Figure 3:**
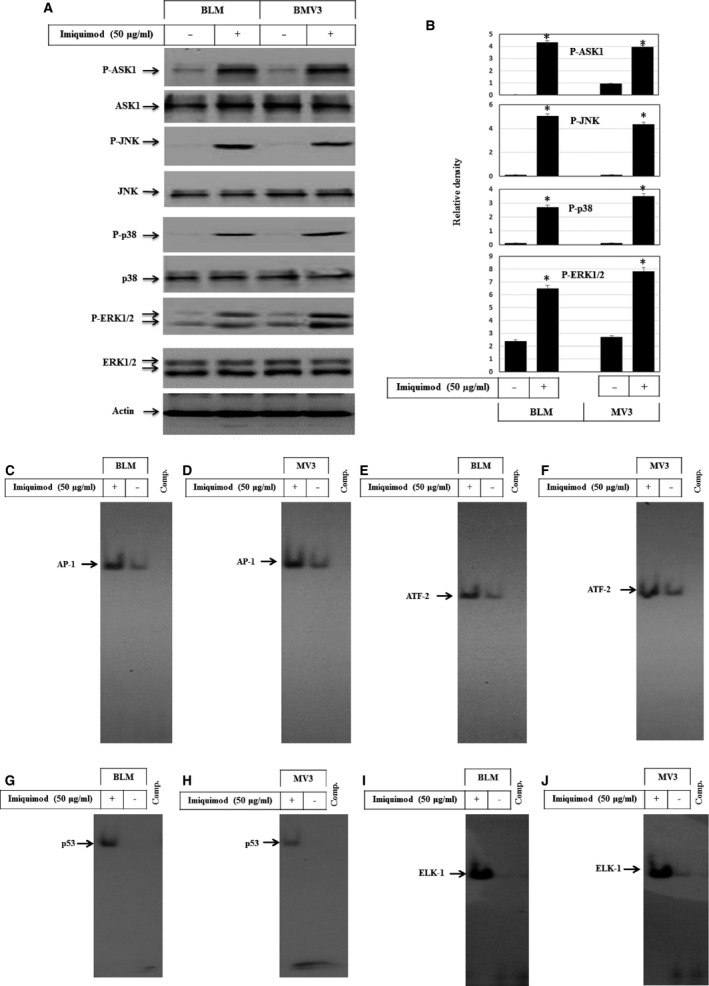
(**A**) Western blot analysis demonstrates the phosphorylation of ASK1, JNK, p38 and ERK1/2 without the alteration of their basal expression in response to the treatment of melanoma cells with imiquimod for 48 hrs. Actin was used as an internal control for loading and transfer. (**B**) Analyses of band intensity on films are presented as the relative ratio of phospho‐PERK, phosphor‐ASK1, phosph‐JNK, phosphor‐p38 and phosphor‐ERK1/2 to actin. Bars represent mean ± SD from three blots. *P < 0.05 *versus* control. EMSA demonstrates the induction of the DNA‐binding activities of the transcription factors AP‐1 (**C** and **D**), ATF‐2 (**E** and **F**), p53 (**G** and **H**) and ELK‐1 (**I** and **J**) in BLM and MV3 cells before and after the exposure to imiquimod for 48 hrs. Data are representative of three independent experiments.

To show whether imiquimod‐induced apoptosis is associated with alterations of the mitogen‐activated protein kinase (MAPK) pathways in melanoma cells, both melanoma cells lines were investigated for the activation of ASK1, JNK, p38 and extracellular regulated kinase (ERK). Data obtained from Western blot analysis (Fig. 3A) demonstrated imiquimod‐induced phosphorylation of ASK1, JNK, p38 and ERK1/2 without impacting their basal expression. In addition, we analysed the DNA‐binding activity of transcription factors such as AP‐1, ATF2, p53 and ELK‐1, typical substrates for MAPKs 20, 41. As expected, EMSA demonstrated the enhanced DNA‐binding activity of the different transcription factors AP‐1 (Fig. 3C and D), ATF‐2 (Fig. 3E and F), p53 (Fig. 3G and H) and ELK‐1 (Fig. 3I and J) in both melanoma cell lines BLM and MV3 upon imiquimod treatment.

### Activation of NF‐κB pathway by imiquimod in melanoma cells

**Figure 4:**
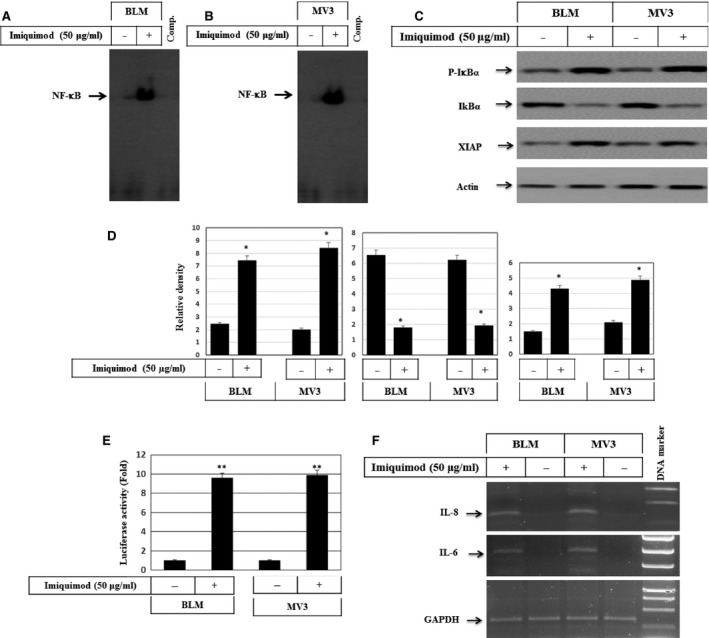
**(A)** Western blot analysis demonstrates the phosphorylation of IκBα and its subsequent degradation and the induction of XIAP in response to the treatment of melanoma cells with imiquimod for 48 hrs. Actin was used as an internal control for loading and transfer. EMSA demonstrates the activation of the nuclear transcription factor NF‐κB in BLM **(B)** and MV3**(C)** melanoma cells in response to the treatment with imiquimod. Data are representative of three independent experiments yielding similar results. **(D)** Analyses of band intensity on films are presented as the relative ratio of phospho‐IκBα, and expression of IκBα, XIAP to actin. Bars represent mean ± SD from three blots. *P < 0.05 *versus* control. **(E)** Luciferase reporter assay demonstrates imiquimod‐induced activation of NF‐κB in both melanoma cell lines, BLM and MV3. Data represented as mean ± SD of three independent experiments. **P < 0.01, significantly different when compared with control cells. **(F)** RT‐PCR demonstrates the induction of both IL‐6 and IL‐8 expression, the target genes of NF‐kB in response to the treatment of both BLM and MV3 cells with imiquimod. Data are representative of three independent experiments.

The activation of NF‐κB by ROS is widely documented 42. Thus, we next examined whether NF‐κB is activated in response to imiquimod‐induced ROS accumulation. As demonstrated by EMSA (Fig. 4A and B), imiquimod induced the DNA‐binding activity of NF‐κB. In line, phosphorylation and degradation of the inhibitor of NF‐κB (IκB)α confirmed the activation of the NF‐κB pathway in response to imiquimod treatment (Fig. 4C and D). Also, analysis of XIAP, the target of the NF‐κB pathway, revealed the induction of XIAP upon imiquimod treatment (Fig. 4C and D). Next, we analysed imiquimod‐induced NF‐κB activation using an NF‐κB reporter assay. Imiquimod increased NF‐κB reporter activity in melanoma cells, when compared to control cells (Fig. 4E). To further confirm these findings, we examined the NF‐kB target gene expression. RT‐PCR was performed for mRNA expression of IL‐6 and IL‐8 (CXCL8). As shown in Figure 4F induction of both IL‐6 and IL‐8 expression in response to treatment of BLM and MV3 cells with imiquimod was found. Taken together, these data demonstrate the ability of imiquimod to trigger the NF‐κB pathway and its effector genes in melanoma cells.

### Imiquimod‐induced ROS accumulation results in the activation of ASK1‐JNK/p38 and NF‐κB pathways

**Figure 5:**
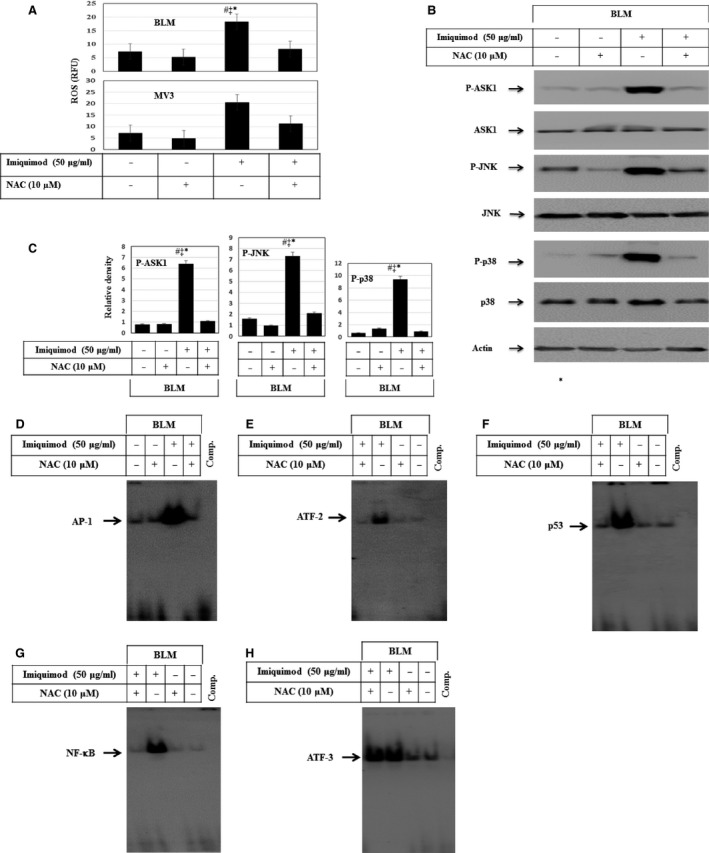
(**A**) Flow cytometry analysis demonstrates the inhibition of imiquimod‐induced ROS accumulation in response to the pre‐treatment of melanoma cell lines BLM and MV3 with the scavenger of ROS (NAC). Data represented as mean ± SD of three independent experiments. *P < 0.05 *versus* control, ^‡^ *P* < 0.05 *versus* NAC, ^#^ *P* < 0.05 *versus* NAC + imiquimod. (**B**) Demonstrates the inhibition of imiquimod‐induced phosphorylation of ASK1, JNK and p38 in BLM cells in response to the inhibition of ROS accumulation by NAC without to influence the basal expression. Actin was used as an internal control for loading and transfer. (**C**) Analyses of band intensity on films are presented as the relative ratio of phospho‐ASK1, phosphor‐JNK and phospho‐p38 to actin. Bars represent mean ± SD from three blots. *P < 0.05 *versus* control, ^‡^ *P* < 0.05 *versus* NAC, ^#^ *P* < 0.05 *versus* NAC + imiquimod. EMSA demonstrates the inhibition of imiquimod‐induced activation of the transcription factors AP‐1 (**D**), ATF‐2 (**E**), p53 (**F**) and NF‐κB (**G**), but not those of the transcription factor ATF‐3 (**H**) in response to the pre‐treatment of the melanoma cell line BLM with NAC. Results are representative of two independent experiments with identical results.

To address the mechanism of how imiquimod triggers the activation of ASK1‐JNK/p38, we examined whether the inhibition of ROS accumulation impacts the imiquimod‐enhanced activation of ASK1 and its downstream pathways. Cells were pre‐treated with N‐Acetyl Cysteine (NAC), the scavenger of ROS, 1 hr prior to the exposure to imiquimod. Flow cytometry analysis of ROS accumulation and Western blot analysis were performed. Figure 5A demonstrates that NAC largely inhibits imiquimod‐induced ROS accumulation. Moreover, data of Western blot analysis (Fig. 5B and C) demonstrate that NAC largely inhibits imiquimod‐induced phosphorylation of ASK1, JNK and p38 in BLM cells. Similar results were noted in MV3 cells (data not shown). In addition, we investigated whether pre‐treatment of melanoma cells with NAC inhibits imiquimod‐induced DNA‐binding activity of the transcription factors AP‐1, ATF‐2, ATF‐3 and p53 as a consequence of the inhibition of ASK1. Electrophoretic mobility shift assay analysis demonstrated the inhibition of imiquimod‐induced AP‐1 (Fig. 5D), ATF‐2 (Fig. 5E), p53 (Fig. 5F) and NF‐κB (Fig. 5G) without affecting imiquimod‐induced DNA‐binding activity of ATF‐3 (Fig. 5H) in BLM cells, suggesting an essential role for ROS accumulation in the modulation of imiquimoid‐induced activation of the transcription factors ATF‐2, p53 and NF‐κB, but not those of ATF‐3.

### Imiquimod‐induced expression of Noxa is mediated by PERK‐IRE1α and ASK1‐JNK pathways

**Figure 6:**
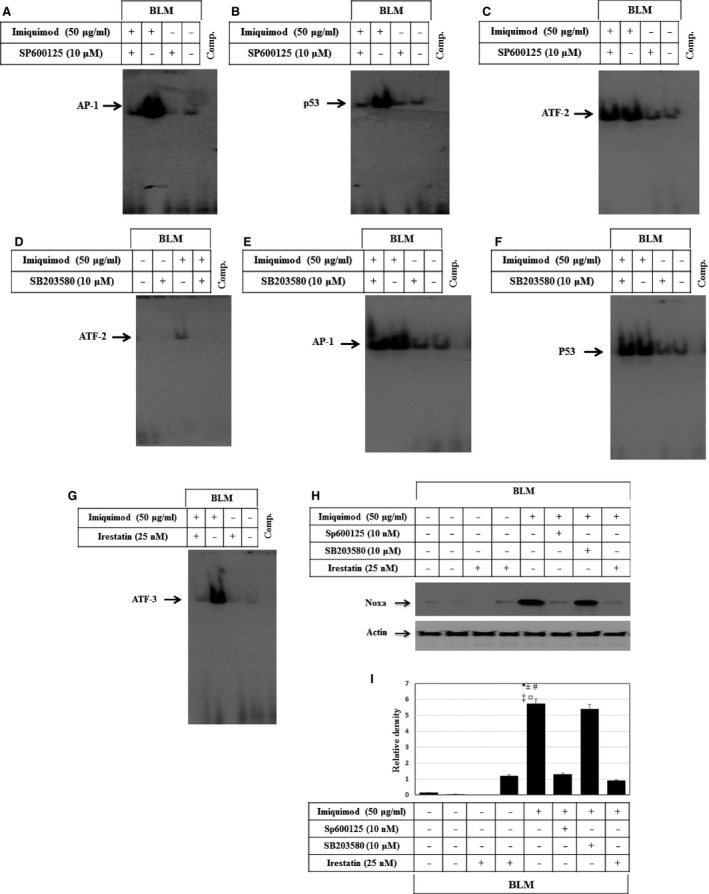
The activation of ASK1‐JNK and PERK‐IRE1α pathways are essential for the modulation of imiquimod‐induced expression of Noxa protein in melanoma cells. EMSA demonstrates that the pre‐treatment of melanoma cell line BLM with the inhibitor of JNK (SP600125) inhibits imiquimod‐ induced DNA‐binding activity of the transcription factors AP‐1 (**A**) and p53 (**B**), but not the induced DNA‐binding activity of the transcription factor ATF‐2 (**C**). Whereas, the pre‐treatment of the melanoma cell line BLM with the inhibitor of p38 (SB203580) inhibits imiquimod‐induced activity of the transcription factor ATF‐2 (**D**), but not those of the transcription factors AP‐1 (**E**) or p53 (**F**). (**G**) EMSA demonstrates the inhibition of imiquimod‐induced DNA‐binding activity of the transcription factor ATF‐3 in response to the pre‐treatment of the melanoma cell line BLM with the inhibitor of IRE1α (irestatin). (**H**) Western blot analysis demonstrates the inhibition of imiquimod‐induced expression of Noxa in response to the pre‐treatment of BLM cells with the inhibitor of JNK or IRE1α, but not by the inhibitor of p38. Actin is used as an internal control for loading and transfer. Data are representative of three independent experiments. (**I**) Analyses of band intensity on films are presented as the relative ratio of phospho‐IκBα, and expression of IκBα, XIAP to actin. Bars represent mean ± SD from three blots. *P < 0.05 *versus* control. ^*#*^ *P* < 0.05 *versus* SP600125, ^‡^ *P* < 0.05 *versus* Irestatin, ^¤^ *P* < 0.05 *versus* SP600125 + imiquimod, ^±^ *P* < 0.05 *versus* Irestatin + imiquimod.

To pinpoint the signalling pathways, responsible for imiquimod‐induced expression of Noxa, both melanoma cell lines BLM and MV3 were treated with imiquimod, when pre‐treated with the inhibitors of JNK (SP600125), p38 (SB203580) and IRE1α (irestatin) 1 hr before treatment with imiquimod. Cells were harvested 48 hrs later to examine the effect of the above mentioned inhibitors. Electrophoretic mobility shift assay demonstrated the inhibition of imiquimod‐induced DNA‐binding activity of the transcription factors AP‐1 (Fig. 6A) and p53 (Fig. 6B), but not of the transcription factor ATF‐2 (Fig. 6C). Moreover, inhibition of the p38 resulted in complete abrogation of imiquimod‐induced ATF‐2 activation (Fig. 6D), but not those of the transcription factors AP‐1(Fig. 6E) or p53 (Fig. 6F). Furthermore, pre‐treatment of melanoma cells with the inhibitor of IRE1α (irestatin) blocked imiquimod‐induced ATF‐3 (Fig. 6G). Using the same three inhibitors, we wanted to study how these imiquimod‐induced signals regulate Noxa protein expression. Analysis of Noxa expression by Western blot (Fig. 6H and I) demonstrated that inhibition of JNK or IRE1α, but not the p38, interferes with imiquimod‐induced expression of Noxa. Of note, similar results were observed in MV3 cells (data not shown). Taken together our data suggest the involvement of AP‐1, p53 and ATF‐3, but not ATF‐2 in the promotion of imiquimod‐induced expression of Noxa protein. Thus, we firmly believe that both PERK‐IRE1α and ASK1‐JNK pathways are involved in the induction of imiquimod‐induced Noxa expression.

### Imiquimod‐induced apoptosis of melanoma cells is mediated by PERK and ASK1 pathways, and enhanced by inhibition of the NF‐κB pathway

**Figure 7:**
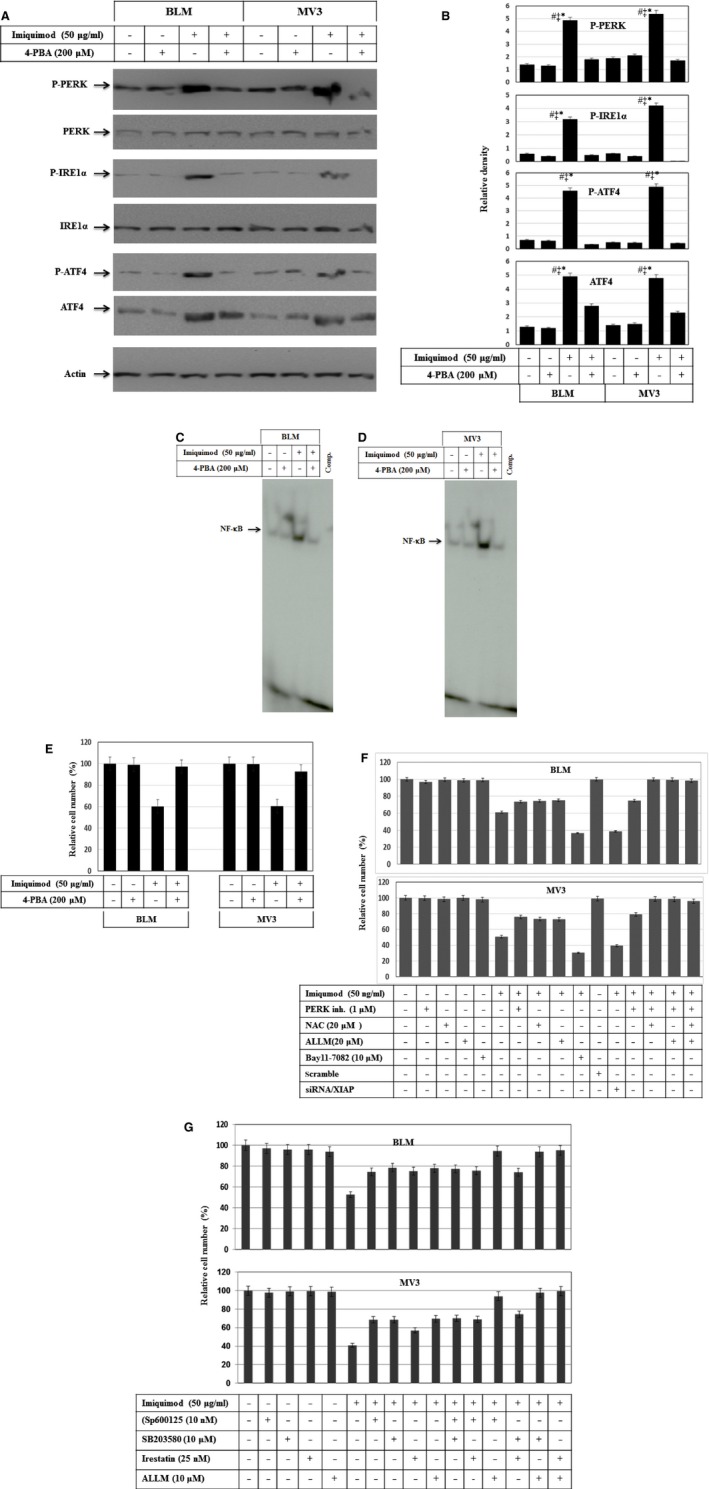
(**A**) Western blot analysis demonstrates the inhibition of imiquimod‐induced phosphorylation of PERK and IRE1α, the expression and phosphorylation of ATF‐4 in melanoma cell lines in response to the treatment with 4‐PBA, the inhibitor of ER stress. (**B**) Analyses of band intensity on films are presented as the relative ratio of phospho‐PERK, phospho‐IRE1α, phospho‐ATF4 and ATF4 to actin. Bars represent mean ± SD from three blots. *P < 0.05 *versus* control. ^#^ *P* < 0.05 *versus* 4‐PBA, ^‡^ *P* < 0.05 *versus* 4‐PBA+imiquimod. EMSA demonstrates the inhibition of imiquimod‐induced NF‐κB activation in BLM (**C**) and MV3 (**D**) cell lines in response to the treatment with 4‐PBA. (**E**) MTT assay demonstrates the abrogation of imiquimod‐induced death of both BLM and MV3 cells in response to the treatment with 4‐PBA, the inhibitor of ER stress. (**F**) MTT assay demonstrates the inhibition of imiquimod‐induced cell death, in part, by the pre‐treatment of melanoma cell lines BLM and MV3 with the inhibitors of PERK (GSK2606414), calpain (ALLM), ROS (NAC) and completely by the combination of the inhibitors of PERK, ROS with the inhibitor of calpain, whereas, the pre‐treatment of melanoma cells with inhibitor of NF‐κB pathway (Bay11‐7082) or the knockdown of XIAP by its specific siRNA enhance imiquimod‐induced apoptosis. (**G**) MTT assay demonstrated the inhibition of imiquimod‐induced cell death, in part, by the pre‐ treatment of melanoma cells with the inhibitors of JNK (SP600125), calpain (ALLM) or IRE1α (irestatin), but not with the inhibitor of p38 (SB203580). Whereas, the combination of the inhibitor of caplain with either JNK or IRE1α inhibitors results in the abrogation of imiquimod‐induced apoptosis of melanoma cell lines BLM or MV3. Data presented are the mean ± SD of three independent experiments performed in duplicate.

To investigate if the inhibition of ER stress influences imiquimod‐induced cytotoxic effects in melanoma cells, we next pre‐treated melanoma cell lines BLM and MV3 with the inhibitor of ER stress 4‐PBA, before exposure to imiquimod 48 hrs after stimulation with imiquimod, cells were assayed for ER stress markers PEPK and its downstream signalling pathways. Expression and phosphorylation of PERK, IRE1α and ATF‐4 were analysed using Western blot (Fig. 7A) and demonstrated substantial inhibition of imiquimod‐induced phosphorylation of PERK, IRE1α and expression and phosphorylation of ATF‐4 by 4‐PBA (Fig. 7A and B). Treatment with 4‐PBA also blocked imiquimod‐induced NF‐κB activity in both BLM (Fig. 7C) and MV3 (Fig. 7D) as demonstrated by EMSA. More importantly, treatment with 4‐PBA rescued imiquimod‐induced cell death of BLM and MV3 cells (Fig. 7E), suggesting that imiquimod‐induced melanoma cell death is mediated *via* ER stress‐dependent mechanism(s).

To determine which of the pathways are implicated in the modulation of imiquimod‐induced apoptosis, melanoma cells were pre‐treated with inhibitors of PERK (GSK2606414), IRE1α (irestatin), ROS (NAC), JNK (SP600125), p38 (SB203580), calpain (ALLM) and NF‐κB (Bay11‐7082) or transfected with XIAP‐specific siRNA before exposure to imiquimod. Although the inhibitors did not influence cellular viability, the inhibition of JNK, PERK, calpain or ROS accumulation by the corresponding inhibitors was found to partially block imiquimod‐induced cell death in both BLM and MV3 melanoma cells (Fig. 7F). In contrast, the inhibition of NF‐κB pathway by Bay11‐7082 or knockdown of XIAP enhanced imiquimod‐induced cell death (Fig. 7F). Although the combination of PERK inhibitor with ALLM was found to partially block imiquimod‐induced cell death, the combination of PERK, NAC and ALLM together completely abrogated imiquimod‐induced cell death (Fig. 7F). Next, we analysed the effect of the inhibitors of JNK, p38, IRE1α and ALLM alone or in combination on imiquimod induced‐apoptosis of melanoma cells. Although the inhibitors of JNK, IRE1α, p38 or calpain did not influence the viability of melanoma cells, pre‐treatment of melanoma cells with inhibitors of JNK, IRE1α and calpain, but not those of p38 partially blocked imiquimod‐induced cell death (Fig. 7G). Only the combination of calpain inhibitor with either those of JNK or IRE1α, resulted in the abrogation of imiquimod‐induced cell death (Fig. 7G). Taken together, the data of the inhibitor experiments suggested an essential role for both PERK‐IRE1 and ASK1‐JNK pathways for imiquimod‐induced apoptosis of melanoma cells.

### Noxa is the main mediator of imiquimod‐induced apoptosis of melanoma cells

**Figure 8:**
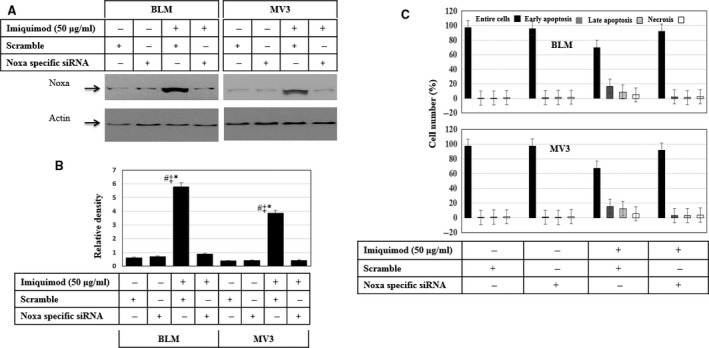
Effect of Noxa knockdown on imiquimod‐induced apoptosis of melanoma cells.** (A)** Western blot analysis demonstrates the efficiency of siRNA for imiquimod‐induced expression of Noxa. Actin was used as an internal control for loading and transfer. **(B)** Analyses of band intensity on films are presented as the relative ratio of Noxa to actin. Bars represent mean ± SD from three blots. *P < 0.05 *versus* control/scremble. ^#^ *P* < 0.05 *versus* siRNA, ^‡^ *P* < 0.05 *versus* siRNA + imiquimod. **(C)** Flow cytometry analysis demonstrates that the knockdown of Noxa expression blocks imiquimod‐induced apoptosis of both melanoma cell lines BLM and MV3. Data are representative of three‐independent experiments performed separately.

To investigate whether Noxa protein is essential for the modulation of imiquimod‐induced apoptosis, we lastly performed knockdown studies. Accordingly, melanoma cell lines BLM and MV3 were transfected with either scrambled or Noxa‐specific siRNA 24 hrs prior to exposure to a 48 hrs imiquimod treatment. Western blot analysis (Fig. 8A and B) revealed the successful knockdown of imiquimod‐induced expression of Noxa in both melanoma cells. Data obtained from flow cytometry analysis revealed that abrogation of Noxa induction fully blocks imiquimod‐induced apoptosis in both BLM and MV3 cells (Fig. 8C). These data firmly establish an essential role for Noxa as an imiquimod‐induced apoptotic mediator critical for imiquimod‐induced melanoma cell apoptosis.

## Discussion

Apoptosis is a highly conserved, innate mechanism by which eukaryotic cells undergo programmed cell‐death. This mechanism permits the elimination of undesired or defective cells by an orderly cascade of cellular disintegration without inducing inflammation 43. In addition to the dysregulated proliferation, substantial evidence indicates that proliferating tumours require anti‐apoptotic mutations to survive and propagate 44. Therefore, the reconstitution of apoptosis in malignant cells by the inhibition of survival pathways is a promising approach in cancer therapy.

Imiquimod belongs to the class of TLR agonists that stimulate both adaptive and innate immunity by interacting with TLR7. This leads to NF‐κB‐mediated transcription of pro‐inflammatory genes in plasmacytoid dendritic cells, including tumor necrosis factor (TNF)‐α, interferon‐α and IL‐12 45, 46. In addition to the activation of NF‐κB pathway 47, imiquimod can trigger apoptosis 48, 49.

In this study, we report for the first time a potential role for ER in the regulation of imiquimod‐induced apoptosis of melanoma cells *via* multiple pathways, initiated by ER stress‐dependent pathways. These pathways firmly established by our study includes imiquimod‐ induced PERK‐IRE1‐ATF‐4‐ATF‐3 activation and in parallel calpain‐caspase‐4‐caspase‐9‐caspase‐3.

In response to ER stress, cells activate UPR leading to the release of the UPR regulator GRP78 protein from ER transmembrane signal transducers including PERK, IRE1α and ATF6. Of note, the release of GRP78 from the ER stress transducers is a pre‐requisite to initiate the activation of UPR transcription and its downstream consequences 50, 51. Thus, if ER stress is prolonged or overwhelming, UPR will fail to maintain normal ER function, and the adaptive UPR will switch to pro‐apoptotic signals, such as CHOP, to eliminate the irreversibly damaged cells 19. Our results showed that the induction of the phosphorylation of PERK and IRE1 α was observed with the expression of CHOP in response to the treatment of melanoma cells with imiquimod, suggesting that imiquimod‐induced ER stress is essential for the modulation of apoptosis.

It has been well‐established that induction of oxidative stress in ER is associated with mitochondrial damage, resulting in excessive production of ROS and subsequent apoptosis 20, 52. In this study, the inhibition of ER stress by 4‐PBA confirmed the association between mitochondria and ER in imiquimod‐treated melanoma cells. 4‐PBA could restore imiquimod‐induced effects on PERK, IRE1, ATF‐4 and NF‐κB together with the inhibition of apoptosis, suggesting that ER acted upstream of mitochondria in imiquimod‐treated cells. Based on our findings, imiquimod‐induced ER stress is involved not only in the modulation of mitochondrial apoptosis but also in the induction of NF‐κB pathways and its downstream effector protein XIAP.

Endoplasmic reticulum plays an important role in the maintenance of intracellular Ca^2+^ homoeostasis. The alteration of Ca^2+^ homoeostasis has been reported to cause the loss of Δψm, cytochrome c release and ROS accumulation 37, as well as the activation of PERK, IRE1α and calpain‐dependent pathways 19.

Our results revealed that the treatment of melanoma cells with imiquimod evokes a rapid increase in intracellular Ca^2+^ release and the activation of PERK and IER1α as well as the degradation of calpain, and anticipates a central role for ER stress‐associated pathways in the modulation of imiquimod‐induced apoptosis of melanoma cells.

Elevation of intracellular calcium can also induce oxidative stress including the uncoupling of mitochondrial respiration and permeability transition 53. Reactive oxygen species, including superoxide anion, H_2_O_2_ and hydroxyl radicals are by‐products of oxidative phosphorylation that are constantly generated during anti‐cancer agent‐induced apoptosis 19, 20. In addition to the induction of apoptosis, ROS can enhance the activation of the NF‐κB 20, 54 and ASK1‐JNK/p38 pathways 55. Accordingly, the activation of the NF‐κB pathway together with the induction of the expression of XIAP protein, the loss of Δψm and ROS accumulation are the consequences of imiquimod‐induced ER stress.

The transcription factor NF‐κB plays an important role in carcinogenesis as well as in the regulation of immune and inflammatory responses 56. In solid tumours like melanomas, NF‐κB activation is a potential consequence of tumour progression‐mediated alteration in the inflammatory microenvironment 57. Accordingly, NF‐κB is expected to be a critical link for the modulation of the crosstalk between inflammation and cancer 58, 59, 60. The activation of NF‐κB during tumour progression has been reported to be associated with the up‐regulation of the tumour promoting cytokines, such as IL‐6 or TNF 61, 62. Moreover, the inhibition of imiquimod‐induced ROS accumulation by NAC or NF‐κB pathway by its specific inhibitor (Bay11‐7082) blocked imiquimod‐induced activation of NF‐κB and subsequently the expression of apoptosis inhibitor XIAP, and enhanced the apoptosis of melanoma.

More importantly, the enhancement of imiquimod‐induced apoptosis by the combination of NF‐κB inhibitor and chemotherapeutic agents may represent a logical alternative approach in melanoma therapy that should be more vigorously tested.

The observation that NF‐κB plays a key role in melanoma cell survival 63, prompted our group to combine the pharmacological agents to assess killing efficiency in melanoma cells. One such agent, Bay 11‐7082, is an irreversible inhibitor of IκB phosphorylation that blocks proteasomal degradation of IκB and retains NF‐κB in the cytoplasm in an inactive state 64. Although interventions that interrupt the NF‐κB pathway may induce cell death by themselves, particularly in cells that depend on ‘tonic’ NF‐κB survival signalling 65, the common NF‐κB inhibitors can only mediate synergistic effects when combined with an appropriate apoptotic stimuli 66. Accumulating evidence has shown that inhibitors of the NF‐κB pathway may also sensitize neoplastic cells to the lethal actions of conventional cytotoxic agents 67. Our present findings suggest that interruption of the NF‐κB cascade in melanoma cells may increase the apoptotic potential of imiquimod.

In this study, we also report that the imiquimod‐induced activation of ASK1 is associated with activation of the downstream signalling pathways JNK and p38. The activation of ASK1 is mediated by imiquimod‐induced ROS accumulation. The activation of ASK1 in response to ROS accumulation is documented in several studies 20, 54, 55. The activation of ASK1‐JNK pathway is considered an essential step in the modulation of imiquimod‐induced apoptosis. This event occurs *via* enhancement of the DNA‐binding activity of the transcription factors AP‐1 and p53 to promote the transcription of Noxa. Interestingly, we found that both PERK‐IRE1α‐ATF‐4 and ASK1‐JNK pathways are essential for imiquimod‐induced Noxa expression *via* activation of transcription factors ATF‐3, and AP‐1 and p53 respectively. The involvement of the PERK‐IRE1α‐ATF‐4 pathway for induction of Noxa protein and the importance of ATF‐3 in the regulation of Noxa have been reported 68, 69. Also, the involvement of ER stress‐induced Ca^2+^‐calpain‐caspase‐4 in the promotion of apoptosis was suggested 19, 70. Thus, in agreement with the above mentioned reports, our results specify an essential role for the Ca^2+^‐calpain‐caspase‐4 pathway for the modulation of imiquimod‐induced apoptosis in melanoma cells.

The potential of the BH3‐only protein Noxa, as a mediator of the mitochondrial apoptosis has been established 37, 71, 72. Up on its induction, Noxa protein localizes to mitochondria and the ER, leading to loss of Δψm and ER stress that subsequently trigger the activation of mitochondrial dysregulation and/or ER stress‐dependent pathways. These signals are, as our study revealed, essential to initiate the apoptotic machinery in melanoma cells. In this study, we demonstrated the subcellular localization of Noxa protein to both mitochondria and ER in response to the treatment with imiquimod, although the induction of Noxa in total cellular lysates makes it difficult to interpret, which signal‐induction or translocation‐is critical for cytotoxicity. In line with a contribution of mitochondria, the loss of Δψm as demonstrated by the increased release of cytochrome c into the cytoplasm, cleavage of caspase‐9, caspase‐3 and PARP, and also the enhancement of ER stress as demonstrated by the increase in intracellular Ca^2+^ release and activation of PERK and IRE1α pathways may contribute to the execution phase of cell death.

**Figure 9:**
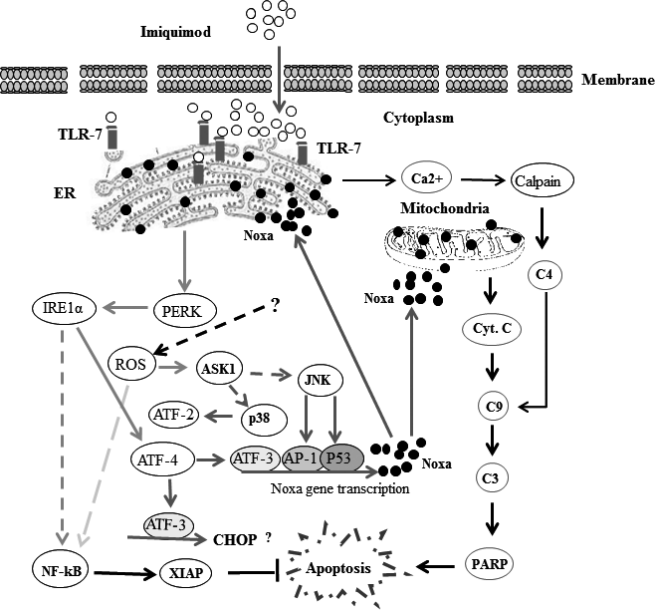
Proposed model for imiquimod‐induced apoptosis of melanoma cells. Binding of imiquimod to TLR7 and/or TLR9 results in the induction of ER stress leading to the activation of PERK, increase of intracellular Ca^2+^ release and accumulation of reactive oxygen species (ROS). The activation of PERK leads to IRE1α activity that, in turn, triggers the activation of NF‐κB pathway and ATF‐4. The activation of NF‐κB pathway results in the expression of the inhibitor of apoptosis, XIAP protein, whereas the activation of ATF‐4 results in the activation of the transcription factor ATF‐3 essential for the transcriptional activation of pro‐apoptotic proteins as well as CHOP. The increased level of cytoplasmic Ca^2+^ results in calpain degradation that subsequently initiates cleavage of caspase‐4, caspase‐9, caspase‐3 and finally PARP. Furthermore, imiquimod‐induced ROS accumulation results in activation of both NF‐κB and ASK1‐JNK/p38 pathways. The activation of ASK1‐JNK/p38 pathway leads to the activation of the transcription factors AP‐1, ATF‐2 and p53. Formation of a transcriptional complex of either AP‐1, p53 and ATF‐3 results in transcriptional activation of Noxa. As a consequence, Noxa localizes on both mitochondria and ER leading to mitochondrial dysregulation and ER stress respectively. The localization of Noxa to ER results in a feedback further increasing ER stress. In turn, mitochondria localization of Noxa triggers the loss of mirochondrial membrane potential (Δψm) and mitochondrial outer membrane permeability (MOMP) characterized by cytochrome c release, cleavage of caspases‐9, caspase‐3 and PARP, altogether evidence for the occurrence of apoptosis.

In summary, we have elucidated the molecular mechanisms that serve as the basis for imiquimod‐induced apoptosis of melanoma cells. In addition, we propose a model outlining the signalling pathways and their key molecules, which are essential for the regulation of imiquimod‐induced apoptosis of melanoma cells (Fig. 9). Accordingly, our findings suggest that imiquimod in combination with NF‐κB inhibitors or IAP antagonists [Geserick *et al*., Cell death and disease 2015; in press] may lead to further advances in melanoma treatment.

## Conflicts of interest

The authors declared that there is no conflict of interest. Robert T. Brodell, M.D., discloses the following potential conflicts of interest: honoraria have been received from presentations for Allergan, Galderma Laboratories, L.P, and PharmaDerm, a division of Nycomed US Inc. Consultant fees have been received from Galderma Laboratories, L.P and Hoffman LaRoche. Clinical trials have been performed for Genentech and Janssen Biotech, Inc.

## References

[1] Kemper K , Krijgsman O , Cornelissen‐Steijger P , *et al* Intra‐ and inter‐tumor heterogeneity in a vemurafenib‐resistant melanoma patient and derived xenografts. EMBO. 2015; 7: 1104–18. Doi:10.15252/emmm.201404914. 10.15252/emmm.201404914 PMC4568946 26105199

[2] Partl R , Fastner G , Kaiser J , *et al* KPS/LDH index: a simple tool for identifying patients with metastatic melanoma who are unlikely to benefit from palliative whole brain radiotherapy. Support Care Cancer. 2015; Doi:10.1007/s00520‐015‐2793‐7. 10.1007/s00520-015-2793-7 26105515

[3] Massi D , Brusa D , Merelli B , *et al* The status of PD‐L1 and tumor‐infiltrating immune cells predict resistance and poor prognosis in BRAFi‐treated melanoma patients harboring mutant BRAFV600. Ann Oncol. 2015; 72: 37–46. 10.1093/annonc/mdv255 26037795

[4] Kessler DA , Austin RH , Levine H . Resistance to chemotherapy: patient variability and cellular heterogeneity. Cancer Res. 2014; 74: 4663–70. 25183790 10.1158/0008-5472.CAN-14-0118

[5] Phadke MS , Sini P , Smalley KS . The novel ATP‐competitive MEK/aurora kinase inhibitor BI‐847325 overcomes acquired BRAF inhibitor resistance through suppression of Mcl‐1 and MEK expression. Mol Cancer Ther. 2015; 14: 1354–64. 25873592 10.1158/1535-7163.MCT-14-0832 PMC4458462

[6] Hornle M , Peters N , Thayaparasingham B , *et al* Caspase‐3 cleaves XIAP in a positive feedback loop to sensitize melanoma cells to TRAIL‐induced apoptosis. Oncogene. 2011; 30: 575–87. 20856198 10.1038/onc.2010.434

[7] Madonna G , Ullman CD , Gentilcore G , *et al* NF‐kappaB as potential target in the treatment of melanoma. J Transl Med. 2012; 10: 53. 22433222 10.1186/1479-5876-10-53 PMC3338086

[8] Geserick P , Herlyn M , Leverkus M . On the TRAIL to overcome BRAF‐inhibitor resistance. J Invest Dermatol. 2014; 134: 315–8. 24424456 10.1038/jid.2013.348

[9] Hwang H , Min H , Kim D , *et al* Imiquimod induces a Toll‐like receptor 7‐independent increase in intracellular calcium *via* IP(3) receptor activation. Biochem Biophys Res Commun. 2014; 450: 875–9. 24971541 10.1016/j.bbrc.2014.06.084

[10] Navi D , Huntley A . Imiquimod 5 percent cream and the treatment of cutaneous malignancy. Dermatol Online J. 2004; 10: 4. 15347486

[11] Ferrandiz L , Ruiz‐de‐Casas A , Trakatelli M , *et al* Assessing physicians’ preferences on skin cancer treatment in Europe. Br J Dermatol. 2012; 167: 29–35. 22881585 10.1111/j.1365-2133.2012.11084.x

[12] Berman B , Poochareon VN , Villa AM . Novel dermatologic uses of the immune response modifier imiquimod 5% cream. Skin Therapy Lett. 2002; 7: 1–6. 12548325

[13] Larange A , Antonios D , Pallardy M , *et al* TLR7 and TLR8 agonists trigger different signaling pathways for human dendritic cell maturation. J Leukoc Biol. 2009; 85: 673–83. 19164127 10.1189/jlb.0808504

[14] Megyeri K , Au WC , Rosztoczy I , *et al* Stimulation of interferon and cytokine gene expression by imiquimod and stimulation by Sendai virus utilize similar signal transduction pathways. Mol Cell Biol. 1995; 15: 2207–18. 7534379 10.1128/mcb.15.4.2207 PMC230449

[15] Blasius AL , Beutler B . Intracellular toll‐like receptors. Immunity. 2010; 32: 305–15. 20346772 10.1016/j.immuni.2010.03.012

[16] Li X , Jiang S , Tapping RI . Toll‐like receptor signaling in cell proliferation and survival. Cytokine. 2010; 49: 1–9. 19775907 10.1016/j.cyto.2009.08.010 PMC2808458

[17] Kim YM , Brinkmann MM , Paquet ME , *et al* UNC93B1 delivers nucleotide‐sensing toll‐like receptors to endolysosomes. Nature. 2008; 452: 234–8. 18305481 10.1038/nature06726

[18] Li ZJ , Sohn KC , Choi DK , *et al* Roles of TLR7 in activation of NF‐kappaB signaling of keratinocytes by imiquimod. PLoS ONE. 2013; 8: e77159. 24146965 10.1371/journal.pone.0077159 PMC3795621

[19] Selimovic D , Ahmad M , El‐Khattouti A , *et al* Apoptosis‐related protein‐2 triggers melanoma cell death by a mechanism including both endoplasmic reticulum stress and mitochondrial dysregulation. Carcinogenesis. 2011; 32: 1268–78. 21693538 10.1093/carcin/bgr112

[20] Selimovic D , Porzig BB , El‐Khattouti A , *et al* Bortezomib/proteasome inhibitor triggers both apoptosis and autophagy‐dependent pathways in melanoma cells. Cell Signal. 2013; 25: 308–18. 23079083 10.1016/j.cellsig.2012.10.004

[21] Bravo R , Gutierrez T , Paredes F , *et al* Endoplasmic reticulum: ER stress regulates mitochondrial bioenergetics. Int J Biochem Cell Bio. 2012; 44: 16–20. 22064245 10.1016/j.biocel.2011.10.012 PMC4118286

[22] Bravo‐Sagua R , Rodriguez AE , Kuzmicic J , *et al* Cell death and survival through the endoplasmic reticulum‐mitochondrial axis. Curr Mol Med. 2013; 13: 317–29. 23228132 10.2174/156652413804810781 PMC4104517

[23] Simmen T , Lynes EM , Gesson K , *et al* Oxidative protein folding in the endoplasmic reticulum: tight links to the mitochondria‐associated membrane (MAM). Biochim Biophys Acta. 2010; 1798: 1465–73. 20430008 10.1016/j.bbamem.2010.04.009 PMC2885528

[24] Thu YM , Su Y , Yang J , *et al* NF‐kappaB inducing kinase (NIK) modulates melanoma tumorigenesis by regulating expression of pro‐survival factors through the beta‐catenin pathway. Oncogene. 2012; 31: 2580–92. 21963849 10.1038/onc.2011.427 PMC3253179

[25] Aguilera‐Aguirre L , Bacsi A , Radak Z , *et al* Innate inflammation induced by the 8‐oxoguanine DNA glycosylase‐1‐KRAS‐NF‐kappaB pathway. J Immunol. 2014; 193: 4643–53. 25267977 10.4049/jimmunol.1401625 PMC4201976

[26] Tang YQ , Jaganath IB , Manikam R , *et al* Inhibition of MAPKs, Myc/Max, NFkappaB, and hypoxia pathways by Phyllanthus prevents proliferation, metastasis and angiogenesis in human melanoma (MeWo) cancer cell line. Int J Med Sci. 2014; 11: 564–77. 24782645 10.7150/ijms.7704 PMC4003541

[27] Lee CH , Jeon YT , Kim SH , *et al* NF‐kappaB as a potential molecular target for cancer therapy. BioFactors. 2007; 29: 19–35. 17611291 10.1002/biof.5520290103

[28] Kucharczak J , Simmons MJ , Fan Y , *et al* To be, or not to be: NF‐kappaB is the answer–role of Rel/NF‐kappaB in the regulation of apoptosis. Oncogene. 2003; 22: 8961–82. 14663476 10.1038/sj.onc.1207230

[29] Luo JL , Kamata H , Karin M . IKK/NF‐kappaB signaling: balancing life and death–a new approach to cancer therapy. J Clin Invest. 2005; 115: 2625–32. 16200195 10.1172/JCI26322 PMC1236696

[30] Papa S , Bubici C , Zazzeroni F , *et al* The NF‐kappaB‐mediated control of the JNK cascade in the antagonism of programmed cell death in health and disease. Cell Death Differ. 2006; 13: 712–29. 16456579 10.1038/sj.cdd.4401865

[31] Liston P , Fong WG , Korneluk RG . The inhibitors of apoptosis: there is more to life than Bcl2. Oncogene. 2003; 22: 8568–80. 14634619 10.1038/sj.onc.1207101

[32] Wright CW , Duckett CS . Reawakening the cellular death program in neoplasia through the therapeutic blockade of IAP function. J Clin Invest. 2005; 115: 2673–8. 16200201 10.1172/JCI26251 PMC1236691

[33] Tang G , Minemoto Y , Dibling B , *et al* Inhibition of JNK activation through NF‐kappaB target genes. Nature. 2001; 414: 313–7. 11713531 10.1038/35104568

[34] Kaur S , Wang F , Venkatraman M , *et al* X‐linked inhibitor of apoptosis (XIAP) inhibits c‐Jun N‐terminal kinase 1 (JNK1) activation by transforming growth factor beta1 (TGF‐beta1) through ubiquitin‐mediated proteosomal degradation of the TGF‐beta1‐activated kinase 1 (TAK1). J Biol Chem. 2005; 280: 38599–608. 16157589 10.1074/jbc.M505671200

[35] Kawakami H , Tomita M , Matsuda T , *et al* Transcriptional activation of survivin through the NF‐kappaB pathway by human T‐cell leukemia virus type I tax. Int J Cancer. 2005; 115: 967–74. 15729715 10.1002/ijc.20954

[36] Shishodia S , Sethi G , Konopleva M , *et al* A synthetic triterpenoid, CDDO‐Me, inhibits IkappaBalpha kinase and enhances apoptosis induced by TNF and chemotherapeutic agents through down‐regulation of expression of nuclear factor kappaB‐regulated gene products in human leukemic cells. Clin Cancer Res. 2006; 12: 1828–38. 16551868 10.1158/1078-0432.CCR-05-2044

[37] Hassan M , Alaoui A , Feyen O , *et al* The BH3‐only member Noxa causes apoptosis in melanoma cells by multiple pathways. Oncogene. 2008; 27: 4557–68. 18408751 10.1038/onc.2008.90

[38] El‐Khattouti A , Selimovic D , Haikel Y , *et al* Identification and analysis of CD133(+) melanoma stem‐like cells conferring resistance to taxol: an insight into the mechanisms of their resistance and response. Cancer Lett. 2014; 343: 123–33. 24080340 10.1016/j.canlet.2013.09.024

[39] Schon MP , Wienrich BG , Drewniok C , *et al* Death receptor‐independent apoptosis in malignant melanoma induced by the small‐molecule immune response modifier imiquimod. J Invest Dermatol. 2004; 122: 1266–76. 15140231 10.1111/j.0022-202X.2004.22528.x

[40] Selimovic D , Hassan M , Haikel Y , *et al* Taxol‐induced mitochondrial stress in melanoma cells is mediated by activation of c‐Jun N‐terminal kinase (JNK) and p38 pathways *via* uncoupling protein 2. Cell Signal. 2008; 20: 311–22. 18068334 10.1016/j.cellsig.2007.10.015

[41] Erhardt A , Hassan M , Heintges T , *et al* Hepatitis C virus core protein induces cell proliferation and activates ERK, JNK, and p38 MAP kinases together with the MAP kinase phosphatase MKP‐1 in a HepG2 Tet‐Off cell line. Virology. 2002; 292: 272–84. 11878930 10.1006/viro.2001.1227

[42] Zeng M , Wei X , Wu Z , *et al* NF‐kappaB‐mediated induction of autophagy in cardiac ischemia/reperfusion injury. Biochem Biophys Res Commun. 2013; 436: 180–5. 23727575 10.1016/j.bbrc.2013.05.070

[43] El‐Khattouti A , Selimovic D , Haikel Y , *et al* Crosstalk between apoptosis and autophagy: molecular mechanisms and therapeutic strategies in cancer. J Cell Death. 2013; 6: 37–55. 25278778 10.4137/JCD.S11034 PMC4147769

[44] Evan GI , Brown L , Whyte M , *et al* Apoptosis and the cell cycle. Curr Opin Cell Biol. 1995; 7: 825–34. 8608013 10.1016/0955-0674(95)80066-2

[45] Urosevic M , Oberholzer PA , Maier T , *et al* Imiquimod treatment induces expression of opioid growth factor receptor: a novel tumor antigen induced by interferon‐alpha? Clin Cancer Res. 2004; 10: 4959–70. 15297396 10.1158/1078-0432.CCR-04-0193

[46] Stary G , Bangert C , Tauber M , *et al* Tumoricidal activity of TLR7/8‐activated inflammatory dendritic cells. J Exp Med. 2007; 204: 1441–51. 17535975 10.1084/jem.20070021 PMC2118597

[47] Schon MP , Schon M , Klotz KN . The small antitumoral immune response modifier imiquimod interacts with adenosine receptor signaling in a TLR7‐ and TLR8‐independent fashion. J Invest Dermatol. 2006; 126: 1338–47. 16575388 10.1038/sj.jid.5700286

[48] Weber A , Kirejczyk Z , Potthoff S , *et al* Endogenous noxa determines the strong proapoptotic synergism of the BH3‐mimetic ABT‐737 with chemotherapeutic agents in human melanoma cells. Transl Oncol. 2009; 2: 73–83. 19412422 10.1593/tlo.08223 PMC2670574

[49] Leverkus M , Gollnick H . “Bak (and Bax) to the future”–of primary melanoma prognosis? J Invest Dermatol. 2006; 126: 1212–4. 16702969 10.1038/sj.jid.5700239

[50] Zhang XY , Zhang TT , Song DD , *et al* Endoplasmic reticulum chaperone GRP78 is involved in autophagy activation induced by ischemic preconditioning in neural cells. Mol Brain. 2015; 8: 20. 25885223 10.1186/s13041-015-0112-3 PMC4381498

[51] Kammoun HL , Chabanon H , Hainault I , *et al* GRP78 expression inhibits insulin and ER stress‐induced SREBP‐1c activation and reduces hepatic steatosis in mice. J Clin Invest. 2009; 119: 1201–15. 19363290 10.1172/JCI37007 PMC2673873

[52] Selimovic D , Badura HE , El‐Khattouti A , *et al* Vinblastine‐induced apoptosis of melanoma cells is mediated by Ras homologous A protein (Rho A) *via* mitochondrial and non‐mitochondrial‐dependent mechanisms. Apoptosis. 2013; 18: 980–97. 23564313 10.1007/s10495-013-0844-4

[53] Hou T , Zhang X , Xu J , *et al* Synergistic triggering of superoxide flashes by mitochondrial Ca^2+^ uniport and basal reactive oxygen species elevation. J Biol Chem. 2013; 288: 4602–12. 23283965 10.1074/jbc.M112.398297 PMC3576066

[54] Cetindere T , Nambiar S , Santourlidis S , *et al* Induction of indoleamine 2,3‐dioxygenase by death receptor activation contributes to apoptosis of melanoma cells *via* mitochondrial damage‐dependent ROS accumulation. Cell Signal. 2010; 22: 197–211. 19799997 10.1016/j.cellsig.2009.09.013

[55] Liang T , Zhang X , Xue W , *et al* Curcumin induced human gastric cancer BGC‐823 cells apoptosis by ROS‐mediated ASK1‐MKK4‐JNK stress signaling pathway. Int J Mol Sci. 2014; 15: 15754–65. 25198898 10.3390/ijms150915754 PMC4200840

[56] Fischer J , Suire C , Hale‐Donze H . Toll‐like receptor 2 recognition of the microsporidia Encephalitozoon spp. induces nuclear translocation of NF‐kappaB and subsequent inflammatory responses. Infect Immun. 2008; 76: 4737–44. 18678660 10.1128/IAI.00733-08 PMC2546815

[57] Bohonowych JE , Hance MW , Nolan KD , *et al* Extracellular Hsp90 mediates an NF‐kappaB dependent inflammatory stromal program: implications for the prostate tumor microenvironment. Prostate. 2014; 74: 395–407. 24338924 10.1002/pros.22761 PMC4306584

[58] Li G , Wang Z , Ye J , *et al* Uncontrolled inflammation induced by AEG‐1 promotes gastric cancer and poor prognosis. Cancer Res. 2014; 74: 5541–52. 25092897 10.1158/0008-5472.CAN-14-0968

[59] Yang T , Zhang X , Wang M , *et al* Activation of mesenchymal stem cells by macrophages prompts human gastric cancer growth through NF‐kappaB pathway. PLoS ONE. 2014; 9: e97569. 24824968 10.1371/journal.pone.0097569 PMC4019592

[60] Wang L , Zhao Y , Liu Y , *et al* IFN‐gamma and TNF‐alpha synergistically induce mesenchymal stem cell impairment and tumorigenesis *via* NFkappaB signaling. Stem Cells. 2013; 31: 1383–95. 23553791 10.1002/stem.1388 PMC3706580

[61] Kim MJ , Yoo YC , Kim HJ , *et al* Aged black garlic exerts anti‐inflammatory effects by decreasing no and proinflammatory cytokine production with less cytoxicity in LPS‐stimulated raw 264.7 macrophages and LPS‐induced septicemia mice. J Med Food. 2014; 17: 1057–63. 25238199 10.1089/jmf.2013.3043

[62] Duo CC , Gong FY , He XY , *et al* Soluble calreticulin induces tumor necrosis factor‐alpha (TNF‐alpha) and interleukin (IL)‐6 production by macrophages through mitogen‐activated protein kinase (MAPK) and NFkappaB signaling pathways. Int J Mol Sci. 2014; 15: 2916–28. 24566135 10.3390/ijms15022916 PMC3958890

[63] Berenson JR , Ma HM , Vescio R . The role of nuclear factor‐kappaB in the biology and treatment of multiple myeloma. Semin Oncol. 2001; 28: 626–33. 11740821 10.1016/s0093-7754(01)90036-3

[64] Pierce JW , Schoenleber R , Jesmok G , *et al* Novel inhibitors of cytokine‐induced IkappaBalpha phosphorylation and endothelial cell adhesion molecule expression show anti‐inflammatory effects *in vivo* . J Biol Chem. 1997; 272: 21096–103. 9261113 10.1074/jbc.272.34.21096

[65] Keller SA , Schattner EJ , Cesarman E . Inhibition of NF‐kappaB induces apoptosis of KSHV‐infected primary effusion lymphoma cells. Blood. 2000; 96: 2537–42. 11001908

[66] Mathieu J , Flexor M , Lanotte M , *et al* A PARP‐1/JNK1 cascade participates in the synergistic apoptotic effect of TNFalpha and all‐trans retinoic acid in APL cells. Oncogene. 2008; 27: 3361–70. 18084321 10.1038/sj.onc.1210997

[67] Patel NM , Nozaki S , Shortle NH , *et al* Paclitaxel sensitivity of breast cancer cells with constitutively active NF‐kappaB is enhanced by IkappaBalpha super‐repressor and parthenolide. Oncogene. 2000; 19: 4159–69. 10962577 10.1038/sj.onc.1203768

[68] Fribley AM , Evenchik B , Zeng Q , *et al* Proteasome inhibitor PS‐341 induces apoptosis in cisplatin‐resistant squamous cell carcinoma cells by induction of Noxa. J Biol Chem. 2006; 281: 31440–7. 16928686 10.1074/jbc.M604356200

[69] Pietkiewicz S , Sohn D , Piekorz RP , *et al* Oppositional regulation of Noxa by JNK1 and JNK2 during apoptosis induced by proteasomal inhibitors. PLoS ONE. 2013; 8: e61438. 23593480 10.1371/journal.pone.0061438 PMC3623862

[70] Lee MJ , Kee KH , Suh CH , *et al* Capsaicin‐induced apoptosis is regulated by endoplasmic reticulum stress‐ and calpain‐mediated mitochondrial cell death pathways. Toxicology. 2009; 264: 205–14. 19699254 10.1016/j.tox.2009.08.012

[71] Saha MN , Jiang H , Yang Y , *et al* PRIMA‐1Met/APR‐246 displays high antitumor activity in multiple myeloma by induction of p73 and Noxa. Mol Cancer Ther. 2013; 12: 2331–41. 24030633 10.1158/1535-7163.MCT-12-1166

[72] Kremer KN , Peterson KL , Schneider PA , *et al* CXCR4 chemokine receptor signaling induces apoptosis in acute myeloid leukemia cells *via* regulation of the Bcl‐2 family members Bcl‐XL, Noxa, and Bak. J Biol Chem. 2013; 288: 22899–914. 23798675 10.1074/jbc.M113.449926 PMC3743469

